# Supplementary material for: Chronic Inflammatory Enteropathy and Low-Grade Intestinal T-Cell Lymphoma Are Associated with Altered Microbial Tryptophan Catabolism in Cats
Source: Animals (Basel). 2023 Dec 23;14(1):67. doi: 10.3390/ani14010067 (PMC10777963; doi:10.3390/ani14010067)
Supplement: Supplementary file 1 [file animals-14-00067-s001.zip › S3File.docx]

**Supplemental Table 1:** Serum concentrations of tryptophan derivatives from cats with chronic inflammatory enteropathy and low-grade intestinal T-cell lymphoma were compared among cats with and without (none) histopathologic evidence of pancreatitis, inflammatory liver infiltrates, and combinations thereof. P-values were derived from Kruskal-Wallis tests and values are presented as medians, interquartile ranges (IQR), maximum (max), and minimum (min). Data are derived from the subset of cats in which histopathologic analysis of the small intestines, pancreata, and livers were available.

|  | **None (N=31)** | **Pancreatitis (N=2)** | **Inflammatory Liver Disease (N=6)** | **Inflammatory Liver Disease and Pancreatitis (N=3)** | **Kruskal-Wallis**  **P-Value** |
| --- | --- | --- | --- | --- | --- |
| **Indoleacetate (ng/mL)** |  |  |  |  | 0.143 |
| Median | 249.400 | 1232.550 | 423.050 | 302.200 |  |
| IQR | 207.250, 334.250 | 875.475, 1589.625 | 267.075, 876.775 | 207.750, 346.300 |  |
| Min | 68.800 | 518.400 | 131.200 | 113.300 |  |
| Max | 3219.300 | 1946.700 | 1528.300 | 390.400 |  |
| **Indolepropionate (ng/mL)** |  |  |  |  | 0.370 |
| Median | 32.700 | 21.600 | 52.850 | 16.100 |  |
| IQR | 23.800, 49.450 | 17.950, 25.250 | 18.775, 86.175 | 13.950, 26.950 |  |
| Min | 11.200 | 14.300 | 15.300 | 11.800 |  |
| Max | 601.700 | 28.900 | 88.500 | 37.800 |  |
| **Indoleacetamide (ng/mL)** |  |  |  |  | 0.230 |
| Median | 1.400 | 1.400 | 1.450 | 1.400 |  |
| IQR | 1.400, 1.400 | 1.400, 1.400 | 1.400, 1.500 | 1.400, 1.500 |  |
| Min | 1.300 | 1.400 | 1.400 | 1.400 |  |
| Max | 2.100 | 1.400 | 1.500 | 1.600 |  |
| **Indoleacrylate (ng/mL)** |  |  |  |  | 0.302 |
| Median | 512.700 | 369.150 | 574.650 | 545.300 |  |
| IQR | 457.600, 674.300 | 335.825, 402.475 | 504.425, 582.175 | 544.900, 546.100 |  |
| Min | 240.300 | 302.500 | 458.300 | 544.500 |  |
| Max | 934.700 | 435.800 | 798.200 | 546.900 |  |
| **Indolelactate (ng/mL)** |  |  |  |  | 0.110 |
| Median | 131.000 | 50.000 | 139.800 | 81.800 |  |
| IQR | 111.950, 247.650 | 49.850, 50.150 | 80.675, 252.925 | 77.150, 205.050 |  |
| Min | 56.000 | 49.700 | 72.400 | 72.500 |  |
| Max | 691.700 | 50.300 | 495.500 | 328.300 |  |
| **Indolepyruvate (ng/mL)** |  |  |  |  | 0.450 |
| Median | 1128.300 | 1135.100 | 1135.800 | 1112.700 |  |
| IQR | 1107.500, 1136.000 | 1127.150, 1143.050 | 1116.700, 1159.325 | 1106.000, 1119.500 |  |
| Min | 1095.700 | 1119.200 | 1108.400 | 1099.300 |  |
| Max | 1230.800 | 1151.000 | 1387.200 | 1126.300 |  |
| **Indolecarboxyaldehyde (ng/mL)** |  |  |  |  | 0.252 |
| Median | 23.300 | 15.450 | 24.900 | 24.800 |  |
| IQR | 19.950, 29.850 | 13.775, 17.125 | 20.975, 26.200 | 24.250, 25.300 |  |
| Min | 8.300 | 12.100 | 18.900 | 23.700 |  |
| Max | 42.300 | 18.800 | 32.700 | 25.800 |  |
| **Kynurenine (µg/mL)** |  |  |  |  | 0.102 |
| Median | 1.018 | 1.404 | 1.552 | 0.988 |  |
| IQR | 0.740, 1.295 | 1.301, 1.508 | 1.301, 2.813 | 0.932, 1.013 |  |
| Min | 0.513 | 1.198 | 0.580 | 0.876 |  |
| Max | 9.811 | 1.611 | 3.791 | 1.038 |  |
| **Kynurenate (ng/mL)** |  |  |  |  | 0.547 |
| Median | 9.300 | 8.300 | 13.150 | 7.900 |  |
| IQR | 6.300, 13.400 | 7.900, 8.700 | 10.700, 42.075 | 7.450, 8.800 |  |
| Min | 4.400 | 7.500 | 3.700 | 7.000 |  |
| Max | 136.200 | 9.100 | 56.300 | 9.700 |  |
| **Serotonin (ng/mL)** |  |  |  |  | 0.194 |
| Median | 760.700 | 1673.850 | 504.900 | 1239.200 |  |
| IQR | 610.900, 950.450 | 1475.025, 1872.675 | 207.200, 1653.850 | 717.350, 1240.350 |  |
| Min | 120.400 | 1276.200 | 12.800 | 195.500 |  |
| Max | 2385.100 | 2071.500 | 3214.100 | 1241.500 |  |
| **Tryptamine (ng/mL)** |  |  |  |  | 0.194 |
| Median | 3.400 | 4.450 | 2.900 | 3.800 |  |
| IQR | 3.150, 3.600 | 4.275, 4.625 | 2.475, 4.375 | 3.250, 3.850 |  |
| Min | 2.500 | 4.100 | 2.300 | 2.700 |  |
| Max | 4.900 | 4.800 | 6.300 | 3.900 |  |
| **Tryptophan (µg/mL)** |  |  |  |  | 0.258 |
| Median | 9.993 | 6.890 | 10.274 | 10.514 |  |
| IQR | 8.775, 12.495 | 6.131, 7.650 | 9.393, 10.863 | 10.366, 10.627 |  |
| Min | 4.164 | 5.371 | 8.413 | 10.218 |  |
| Max | 16.898 | 8.410 | 14.007 | 10.741 |  |

**Supplemental Table 2:** Serum concentrations of tryptophan derivatives from cats with chronic inflammatory enteropathy and low-grade intestinal T-cell lymphoma were compared among cats with and without (none) previous diagnoses of chronic kidney disease (CKD), hyperthyroidism, and combinations thereof. P-values were derived from Kruskal-Wallis tests and values are presented as medians, interquartile ranges (IQR), maximum (max), and minimum (min). Data are derived from the subset of cats in which medical records contained sufficient information to assess for these previous diagnoses.

|  | **None (N=54)** | **CKD (N=15)** | **Hyperthyroidism (N=4)** | **CKD and Hyperthyroidism (N=2)** | **P-value** |
| --- | --- | --- | --- | --- | --- |
| **Indoleacetate (ng/mL)** |  |  |  |  | 0.050 |
| Median | 206.450 | 268.000 | 180.350 | 869.150 |  |
| IQR | 118.500, 328.650 | 236.250, 679.850 | 70.150, 347.175 | 539.575, 1198.725 |  |
| Min | 22.100 | 160.600 | 68.800 | 210.000 |  |
| Max | 1520.300 | 3219.300 | 518.400 | 1528.300 |  |
| **Indolepropionate (ng/mL)** |  |  |  |  | 0.278 |
| Median | 28.200 | 28.900 | 21.750 | 55.900 |  |
| IQR | 17.150, 41.825 | 24.650, 162.400 | 15.575, 28.800 | 39.800, 72.000 |  |
| Min | 3.600 | 12.700 | 14.300 | 23.700 |  |
| Max | 268.500 | 601.700 | 32.700 | 88.100 |  |
| **Indoleacetamide (ng/mL)** |  |  |  |  | 0.018 |
| Median | 1.400 | 1.400 | 1.350 | 1.350 |  |
| IQR | 1.400, 1.400 | 1.400, 1.500 | 1.300, 1.400 | 1.325, 1.375 |  |
| Min | 1.300 | 1.400 | 1.300 | 1.300 |  |
| Max | 2.000 | 2.100 | 1.400 | 1.400 |  |
| **Indoleacrylate (ng/mL)** |  |  |  |  | 0.722 |
| Median | 549.050 | 538.900 | 480.050 | 519.150 |  |
| IQR | 460.200, 761.300 | 471.350, 617.550 | 450.725, 557.775 | 489.325, 548.975 |  |
| Min | 165.000 | 293.700 | 435.800 | 459.500 |  |
| Max | 1239.300 | 934.700 | 717.900 | 578.800 |  |
| **Indolelactate (ng/mL)** |  |  |  |  | 0.618 |
| Median | 136.250 | 124.100 | 95.250 | 111.800 |  |
| IQR | 89.425, 255.550 | 115.100, 186.050 | 82.325, 132.250 | 102.200, 121.400 |  |
| Min | 42.100 | 50.300 | 49.700 | 92.600 |  |
| Max | 495.500 | 691.700 | 237.100 | 131.000 |  |
| **Indolepyruvate (ng/mL)** |  |  |  |  | 0.446 |
| Median | 1118.950 | 1128.300 | 1128.850 | 1108.050 |  |
| IQR | 1108.250, 1140.350 | 1114.100, 1138.100 | 1126.250, 1133.700 | 1107.875, 1108.225 |  |
| Min | 1090.900 | 1095.700 | 1119.200 | 1107.700 |  |
| Max | 1387.200 | 1162.300 | 1147.500 | 1108.400 |  |
| **Indolecarboxyaldehyde (ng/mL)** |  |  |  |  | 0.669 |
| Median | 24.650 | 23.300 | 19.950 | 22.350 |  |
| IQR | 19.475, 32.775 | 20.850, 26.600 | 19.400, 23.650 | 21.575, 23.125 |  |
| Min | 6.400 | 12.100 | 18.800 | 20.800 |  |
| Max | 53.200 | 42.300 | 33.700 | 23.900 |  |
| **Kynurenine (µg/mL)** |  |  |  |  | 0.904 |
| Median | 1.134 | 1.344 | 1.272 | 1.287 |  |
| IQR | 0.991, 1.547 | 0.852, 1.555 | 1.212, 1.378 | 1.162, 1.413 |  |
| Min | 0.513 | 0.617 | 1.198 | 1.037 |  |
| Max | 9.811 | 3.229 | 1.530 | 1.538 |  |
| **Kynurenate (ng/mL)** |  |  |  |  | 0.260 |
| Median | 8.450 | 10.400 | 13.050 | 14.900 |  |
| IQR | 6.325, 13.625 | 7.500, 15.850 | 11.800, 14.675 | 14.350, 15.450 |  |
| Min | 3.700 | 4.400 | 9.100 | 13.800 |  |
| Max | 136.200 | 78.200 | 18.500 | 16.000 |  |
| **Serotonin (ng/mL)** |  |  |  |  | 0.860 |
| Median | 883.950 | 760.700 | 973.150 | 1241.700 |  |
| IQR | 570.050, 1211.050 | 636.950, 1021.600 | 710.700, 1422.750 | 866.800, 1616.600 |  |
| Min | 12.800 | 120.400 | 623.400 | 491.900 |  |
| Max | 3214.100 | 1781.400 | 2071.500 | 1991.500 |  |
| **Tryptamine (ng/mL)** |  |  |  |  | 0.923 |
| Median | 3.500 | 3.400 | 3.500 | 3.850 |  |
| IQR | 3.200, 3.800 | 3.200, 3.800 | 3.200, 4.050 | 3.375, 4.325 |  |
| Min | 2.300 | 2.400 | 3.200 | 2.900 |  |
| Max | 6.300 | 4.500 | 4.800 | 4.800 |  |
| **Tryptophan (µg/mL)** |  |  |  |  | 0.631 |
| Median | 10.627 | 9.993 | 9.152 | 9.306 |  |
| IQR | 8.557, 13.691 | 9.006, 10.745 | 8.928, 10.397 | 8.878, 9.735 |  |
| Min | 3.217 | 5.371 | 8.410 | 8.449 |  |
| Max | 21.474 | 16.898 | 13.980 | 10.164 |  |
