# Supplementary figures and images for: Chronic Inflammatory Enteropathy and Low-Grade Intestinal T-Cell Lymphoma Are Associated with Altered Microbial Tryptophan Catabolism in Cats
Source: Animals (Basel). 2023 Dec 23;14(1):67. doi: 10.3390/ani14010067 (PMC10777963; doi:10.3390/ani14010067)

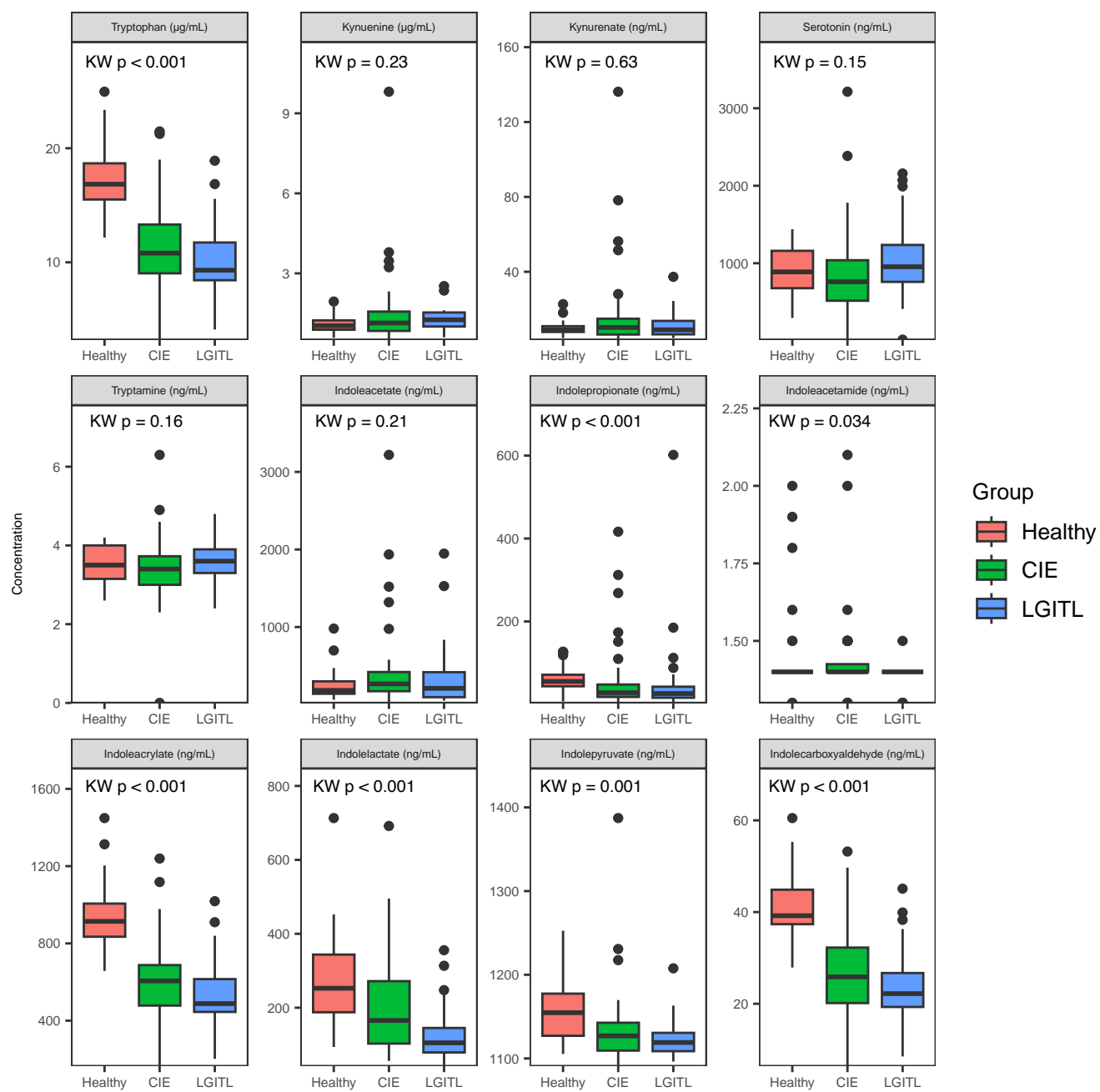

Supplement: Supplementary file 1 [file animals-14-00067-s001.zip › S1File.pdf]
